# The Castrop formula for calculation of toric intraocular lenses

**DOI:** 10.1007/s00417-021-05287-w

**Published:** 2021-07-08

**Authors:** Achim Langenbucher, Nóra Szentmáry, Alan Cayless, Johannes Weisensee, Jascha Wendelstein, Peter Hoffmann

**Affiliations:** 1 Department of Experimental Ophthalmology, Saarland University, Kirrberger Str 100 Bldg. 22, 66424 Homburg/Saar, Germany; 2 Dr. Rolf M. Schwiete Center for Limbal Stem Cell and Aniridia Research, Saarland University, Homburg/Saar, Germany; 3 Department of Ophthalmology, Semmelweis University, Budapest, Hungary; 4 School of Physical Sciences, The Open University, Milton Keynes, UK; 5 Department of Ophthalmology, Johannes Kepler University Linz, Linz, Austria; 6 Augen-und Laserklinik Castrop-Rauxel, Castrop-Rauxel, Germany

**Keywords:** Castrop formula, Toric intraocular lenses, Vergence calculation, Gaussian optics, Prediction of postoperative refraction

## Abstract

**Purpose:**

To explain the concept behind the Castrop toric lens (tIOL) power calculation formula and demonstrate its application in clinical examples.

**Methods:**

The Castrop vergence formula is based on a pseudophakic model eye with four refractive surfaces and three formula constants. All four surfaces (spectacle correction, corneal front and back surface, and toric lens implant) are expressed as spherocylindrical vergences. With tomographic data for the corneal front and back surface, these data are considered to define the thick lens model for the cornea exactly. With front surface data only, the back surface is defined from the front surface and a fixed ratio of radii and corneal thickness as preset. Spectacle correction can be predicted with an inverse calculation.

**Results:**

Three clinical examples are presented to show the applicability of this calculation concept. In the 1st example, we derived the tIOL power for a spherocylindrical target refraction and corneal tomography data of corneal front and back surface. In the 2nd example, we calculated the tIOL power with keratometric data from corneal front surface measurements, and considered a surgically induced astigmatism and a correction for the corneal back surface astigmatism. In the 3rd example, we predicted the spherocylindrical power of spectacle refraction after implantation of any toric lens with an inverse calculation.

**Conclusions:**

The Castrop formula for toric lenses is a generalization of the Castrop formula based on spherocylindrical vergences. The application in clinical studies is needed to prove the potential of this new concept.

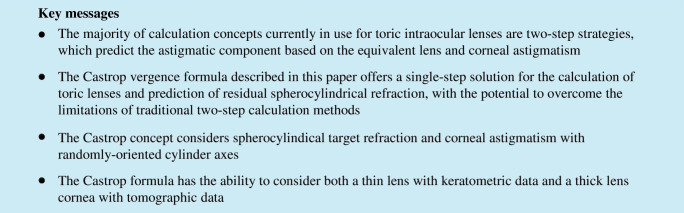

## Background

The first intraocular lens (IOL) power calculation formulae were published in 1967 by Fyodorov [1] and independently in 1970 by Gernet, Ostholt, and Werner [2]. Since then, a wide range of formulae have been proposed by different scientists [1–13]. Some of these calculation concepts are purely empirical, others are so-called theoretical-optical formulae, and others are based on full aperture ray tracing. Empirical concepts include the simple regression formula such as the SRK or SRK2 formula [10, 12], and also modern strategies of machine learning such as the PEARL (Prediction Enhanced Artificial intelligence and output Linearization [7, 11, 13]) or the Hill-RBF (Radial Base Function) calculator. Theoretical-optical formulae are mostly based on paraxial vergence calculation [1, 2, 14] using linear Gaussian optics, where the effective lens position of the lens implant (mostly simplified with a thin lens model) is estimated by empirical means [15–18]. Full aperture ray tracing concepts such as Okulix use tomographic data from the corneal front and back surface together with design data for the IOL front and back surface provided by the IOL manufacturer to trace a bundle of rays from the object to the retinal image plane in order to calculate an appropriate lens power [19–22]. But as with theoretical-optical formulae, the axial position of the IOL has to be estimated empirically prior to cataract surgery [15–18].

With most of the classical calculation concepts, toric intraocular lenses (tIOL) can be easily calculated in 2 separate steps for the two cardinal meridians of the cornea: in the first, we calculate the lens power for the flat corneal meridian, and in the second, the respective lens power is calculated for the steep corneal meridian [14, 23]. This works quite well provided the optical model does not involve crossed cylinders. Once crossed cylinders are involved, this simplification into a 2-step calculation is no longer applicable—for instance, in cases with an arbitrary spherocylindrical target refraction, measurement data from the corneal back surface where the astigmatic axis is not fully aligned to the corneal front surface astigmatism, a surgically induced astigmatism (SIA), or any correction strategy for corneal back surface astigmatism which is not reflected by keratometry [24–30].

As an alternative, if we restrict the model to linear Gaussian optics, we could use spherocylindrical vergences instead of spherical vergences, or equivalently we could use 4 × 4 matrix algebra instead of 2 × 2 matrices to address spherocylindrical surfaces with random axes [31, 32]. Such general concepts allow an unlimited number of refracting surfaces to be considered, and therefore, we could even calculate the appropriate lens power by entering data from a thick lens spectacle correction and a thick lens cornea model, as well as a thick lens IOL. However, in most cases, the respective design data for the spectacle correction will not be available and the design data for the IOL is not provided by the IOL manufacturer. Therefore, the IOL will typically be restricted to a thin lens model until IOL manufacturers are willing to provide shape data for the lens (front and back surface curvature, central thickness, and refractive index for all power steps). In contrast, with modern corneal or anterior segment tomography, the shape data of the corneal front and back surface as well as the corneal thickness can be derived and these could be directly included in the calculation concept.

In principle, such a vergence [14] or matrix calculation [31, 32] with spherocylindrical surfaces is no more complex than the calculation with spherical surfaces. Vergences are traced step-by-step through all surfaces, and the matrix concept yields an en bloc solution, which, in addition, directly provides object to image magnification from the system matrix representing the entire optical system. But both concepts—vergence and matrix—yield identical results for the power of the lens implant or, if we reverse the calculation and consider the power and orientation of the tIOL, for the estimated spherocylindrical refraction at spectacle plane.

The purpose of this study was to show how the Castrop formula as a vergence-based calculation concept with paraxial simplifications with a “thick lens” cornea and a “thin lens toric intraocular lens” could be generalized for prediction of the power of toric lens implants as well as the prediction of postoperative refraction. In this generalization, all surfaces should be considered spherocylindrical with astigmatic axes at random orientations.

## Methods

### The Castrop calculation concept for toric IOL

**Fig. 1:**
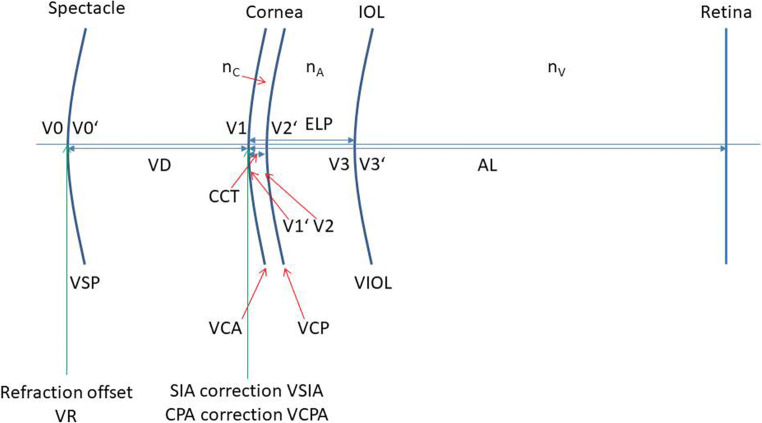
Schematic model for the optical system of the pseudophakic eye. The model is defined by 4 refracting surfaces: a spectacle correction, corneal front and back surface, and intraocular lens. Vx refers to the spherocylindrical vergence in front of, and Vx′ to the spherocylindrical vergence behind, the refractive surface x. The effective lens position (ELP) is derived from the anterior chamber depth and the lens thickness of the phakic eye using formula constants C and H. SIA and CPA refer to the surgically induced astigmatism, both considered at the front apex plane of the cornea, and the refractive correction R (from the formula constant R) is considered at the spectacle plane

The Castrop formula is a vergence formula restricted to linear Gaussian optics in the paraxial space based on a pseudophakic model eye with 4 refractive surfaces: a refractive correction at spectacle plane, a thick cornea with front and back surface, and a thin intraocular lens. The formula considers 3 formula constants (C, H, and R). For long and short eyes, the measured axial length (AL) is transformed to AL_cor_ using a linear regression as described by Cooke et al. [33, 34] with AL_cor_ = 1.23854 + 0.95855·AL-0.05467·LT, where LT refers to the central thickness of the crystalline lens. The effective lens position (ELP) referring to the axial position of the IOL with respect to the corneal front vertex is derived from the external anterior chamber depth (ACD) and the lens thickness (LT) of the phakic eye together with 2 of the 3 formula constants to ELP = ACD + C·LT + H. The third formula constant R is used as an adjustment term for the predicted refraction. The respective optical model for the pseudophakic eye is shown in Fig. 1. All input parameters used for calculation of the toric lens power or prediction of refraction are summarized in Table 1.

**Table 1:** Input parameters of the Castrop formula for toric intraocular lenses. The parameters correspond to the schematics in Fig. 1

Description	Parameters	Units	Comment
Target refraction (for IOL power calculation)	TRS	dpt	Used to define the vergence for spectacle correction VSP
	TRC	dpt	
	TRA	°	
Corneal front surface	RCA_1_	mm	Used to define the vergence for corneal front surface power VCA
	ACA_1_	°	
	RCA_2_	mm	
Corneal back surface (optional)	RCP_1_	mm	Used to define the vergence for corneal back surface power VCP
	ACP_1_	°	
	RCP_2_	mm	
Intraocular lens (for estimation of resulting spectacle refraction)	iIOLS	dpt	Used to define the vergence for implanted intraocular lens ViIOL. According to EN ISO 11979, the spherical equivalent power (iIOLS+0.5·iIOLC) and the absolute cylinder power (iIOLC) are labeled.
	iIOLC	dpt	
	iIOLA	°	
Surgical-induced astigmatism (optional)	SIAC	dpt	Used to define the vergence for surgical-induced astigmatism VSIA
	SIAA	°	
Correction for corneal back surface astigmatism (optional)	CPAC	dpt	Used to define the vergence for statistical correction of the corneal back surface astigmatism VCPA
	CPAA	°	
Constants for the Castrop formula	C	1	Estimation of the ratio of distances (lens equatorial plane − lens front apex / lens thickness in the phakic eye)
	H	mm	Offset correction for the intraocular lens position
	R	dpt	Refraction offset, used to define the vergence for offset in spectacle refraction VR with formula constant R
Distances of the phakic eye	AL	mm	Axial length
	CCT	μm	Central corneal thickness
	ACD	mm	External anterior chamber depth
	LT	mm	Central thickness of the crystalline lens

For the calculation of tIOL, all vergences (V0, V0_, V1, V1_, V2, V2_, V3, and V3_) are considered to be spherocylindric. To simplify the calculation concept, each vergence V is characterized by a vector containing 8 elements. The first 5 elements describe the “standard notation,” whereas the last 3 elements refer to the “component notation” as described previously [14, 27]:

1 $$ \mathrm{V}=\left[{P}_1,{A}_1,{P}_2,{A}_2,\mathrm{AST},\mathrm{EQ},{C}_0,{C}_{45}\ \right] $$

P_1_ and A_1_ refer to the power and axis of the first cardinal meridian (flat or steep), P_2_ and A_2_ to the power and axis of the second cardinal meridian (A_2_ in general orthogonal to A_1_), AST to the difference of refractive power in both cardinal meridians P_2_−P_1_ (positive or negative), EQ to the average power of both meridians (P_1_+P_2_)/2, and C_0_ and C_45_ to the components of AST in terms of a projection to the 0/90° and the 45/135° meridian with C_0_=AST·cos(2·A_1_) and C_45_=AST·sin(2·A_1_).

If we trace through a homogeneous optical medium, the power values P_1_ and P_2_ change according to the vergence transformation formula keeping the axes A_1_ and A_2_ constant [24], and if we consider a spherocylindrical refractive surface, we add together the respective components EQ, C_0_, and C_45_.

For a step-by-step calculation, 2 function modules are required which can be defined as follows:

- Transform_Vergence:

2 $$ \mathrm{Vergence}\_\mathrm{new}:= \left[\frac{P_1}{1-{P}_1\cdotp \frac{d}{n}},{A}_1,\frac{P_2}{1-{P}_2\cdotp \frac{d}{n}},{A}_2,\frac{P_2}{1-{P}_2\cdotp \frac{d}{n}}-\frac{P_1}{1-{P}_1\cdotp \frac{d}{n}},0.5\cdotp \left(\frac{P_2}{1-{P}_2\cdotp \frac{d}{n}}+\frac{P_1}{1-{P}_1\cdotp \frac{d}{n}}\right),\left(\frac{P_2}{1-{P}_2\cdotp \frac{d}{n}}-\frac{P_1}{1-{P}_1\cdotp \frac{d}{n}}\right)\cdotp \cos \left(2\cdotp {A}_1\right),\left(\frac{P_2}{1-{P}_2\cdotp \frac{d}{n}}-\frac{P_1}{1-{P}_1\cdotp \frac{d}{n}}\right)\cdotp \sin \left(2\cdotp {A}_1\right)\right] $$

where P_1_, A_1_, P_2_, and A_2_ correspond to the elements of the vergence before tracing through the homogeneous medium with geometrical thickness d and refractive index n.

- Add_Surface:

3 $$ \mathrm{Vergence}\_\mathrm{new}:= \left[{P}_{1\mathrm{new}},{A}_{1\mathrm{new}},{P}_{2\mathrm{new}},{A}_{1\mathrm{new}},{AST}_{\mathrm{new}}, EQ+{EQ}_{surf}, EQ+{EQ}_{\mathrm{surf}},{C}_0+{C_0}_{\mathrm{surf}},{C}_{45}+{C_{45}}_{\mathrm{surf}}\right] $$

where EQ, C_0_, and C_45_ correspond to the elements of the vergence before adding up the refractive surface, and EQ_surf_, C_0surf_, and C_45surf_ to the respective elements of the refractive surface. The elements of the standard notation after adding up the refracting surface are given by:

4 $$ {\displaystyle \begin{array}{l}{\mathrm{A}\mathrm{ST}}_{\mathrm{new}}=\sqrt{{\left({C}_0+{C_0}_{\mathrm{surf}}\right)}^2+{\left({C}_{45}+{C_{45}}_{\mathrm{surf}}\right)}^2,}\\ {}\begin{array}{l}{\mathrm{P}}_{1\mathrm{new}}=Q+{EQ}_{\mathrm{surf}}-\raisebox{1ex}{$1$}\!\left/ \!\raisebox{-1ex}{$2$}\right.{AST}_{\mathrm{new}},\\ {}\begin{array}{l}{\mathrm{P}}_{2\mathrm{new}}= EQ+{EQ}_{\mathrm{surf}}+\raisebox{1ex}{$1$}\!\left/ \!\raisebox{-1ex}{$2$}\right.{AST}_{\mathrm{new}},\\ {}\begin{array}{l}{\mathrm{A}}_{1\mathrm{new}}=\raisebox{1ex}{$1$}\!\left/ \!\raisebox{-1ex}{$2$}\right.\cdotp {\tan}^{-1}\frac{C_{45}+{C_{45}}_{\mathrm{surf}}}{C_0+{C_0}_{\mathrm{surf}}},\mathrm{and}\\ {}{\mathrm{A}}_{2\mathrm{new}}=90+\raisebox{1ex}{$1$}\!\left/ \!\raisebox{-1ex}{$2$}\right.\cdotp {\tan}^{-1}\frac{C_{45}+{C_{45}}_{\mathrm{surf}}}{C_0+{C_0}_{\mathrm{surf}}}.\end{array}\end{array}\end{array}\end{array}} $$

The refractive indices of the optical model as shown in Fig. 1 are derived from the Liou-Brennan schematic model eye [35] (cornea: n_C_ = 1.376, aqueous humor: n_A_ = 1.336, vitreous: n_V_ = 1.336). The vertex distance (VD) as the distance of the back vertex of the spectacle correction to the corneal front apex is assumed to be 12 mm.

The corneal front surface is defined by 3 parameters, the radius RCA_1_ in an axis of ACA_1_ and the radius RCA_2_ in an axis orthogonal to ACA_1_ (ACA_2_ = ACA_1_ + 90°). For the corneal back surface, the respective parameters are the radius RCP_1_ in an axis of ACP_1_ and the radius RCP_2_ in an axis orthogonal to ACP_1_ (ACP_2_ = ACP_1_ + 90°). If corneal back surface data are not available, the corneal back surface radius is calculated from the respective corneal front surface radius assuming a fixed proportion of front to back surface radius (derived from the Liou-Brennan schematic model eye [35]) with RCP_1_ = RCA_1_·6.4/7.77 (in an axis ACP_1_ = ACA_1_) and RCP_2_ = RCA_2_·6.4/7.77 (in an axis ACP_2_ = ACA_2_). If CCT is not available, we use CCT = 500 μm as extracted from the Liou-Brennan schematic model eye. Corneal front surface power in both cardinal meridians is calculated using PCA_1,2_ = (n_C_-1)/RCA_1,2_, and corneal back surface power using PCP_1,2_ = (n_A_−n_C_)/RCP_1,2_.

For IOL power calculation, the target refraction (TR) at the spectacle plane is expressed in terms of sphere (TRS), cylinder (TRC), and cylinder axis (TRA), either plus or minus cylinder. For prediction of spherocylindrical refraction (PREF) after implantation of the tIOL, the power of the implanted lens is expressed in terms of sphere (iIOLS) or spherical equivalent power (iIOLEQ) and the absolute cylinder power (iIOLC) implanted with an axis (iIOLA).

### Definition of the vergences for the refracting surfaces

For the calculation of the power of a toric lens implant, we calculate the vergence in front of (V3) and behind (V3′) the tIOL. According to Eq. (1) the following parameters: vergence for the target refraction (VTR), both corneal surfaces (VCA and VCP), the surgical astigmatism (VSIA), the adjustment for the corneal back surface astigmatism (VCPA), if measurement is unavailable and the back surface curvature is derived from the corneal front surface [24–30] and the correction for the refraction offset based on the formula constant R, are all defined as follows:

VTR : [TRS, TRA, TRS+TRC, TRA+90°, TRC, TRC+0.5·TRC, TRC·cos(2·TRA), TRC·sin(2·TRA)] for calculation of IOL power,
ViIOL: [iIOLS, iIOLA, iIOLS+iIOLC, iIOLA+90°, iIOLC, iIOLEQ=iIOLC+0.5·iIOLC, iIOLC·cos(2·iIOLA), iIOLC·sin(2·iIOLA)] for prediction of refraction after IOL implantation,
VCA : [PCA_1_, ACA_1_, PCA_2_, ACA_1_+90°, PCA_2_−PCA_1_, 0.5·(PCA_1_+PCA_2_), (PCA_2_−PCA_1_) cos(2·ACA_1_), (PCA_2_−PCA_1_) sin(2·ACA_1_)],
VCP : [PCP_1_, ACP_1_, PCP_2_, ACP_1_+90°, PCP_2_−PCP_1_, 0.5·(PCP_1_+PCP_2_), (PCP_2_−PCP_1_) cos(2·ACP_1_), (PCP_2_−PCP_1_) sin(2·ACP_1_)],
  VSIA
  [−0.5·SIAC, SIAA, 0.5·SIAC, SIAA+90°, SIAC, 0, SIAC·cos(2·SIAA), SIAC·sin(SIAA)],
VCPA : [0.5·CPAC, 90°, −0.5·CPAC, 0°, −CPAC, 0, −CPAC, 0], and
  VR
  [R, 0, R, 90°, 0, R, 0, 0].

### Calculation scheme for the power of a toric lens

**Fig. 2:**
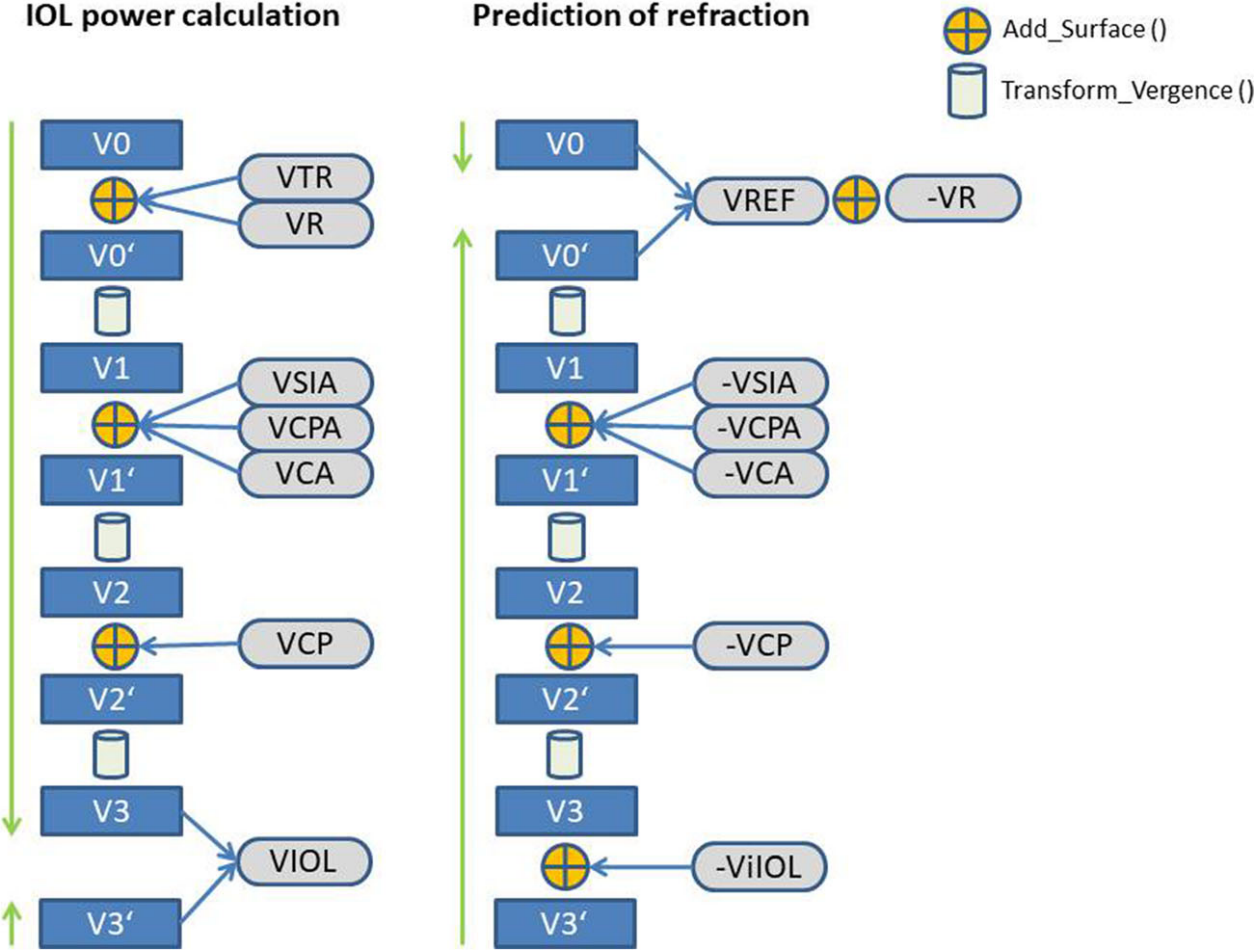
Schematics of the calculation concept for toric intraocular lenses (left) and prediction of the refraction after implantation of a toric lens (right). Add_Surface () and Transform_Vergence () refer to functions of adding up a spherocylindrical surface to a vergence and tracing a spherocylindrical vergence through a homogeneous optical medium. The power of the toric lens implant is calculated from the difference of vergence V3′ and V3 (left), whereas the spherocylindrical refraction at the spectacle plane is derived from the difference of vergence V0′ and vergence (V0 − VR)

The calculation concept for toric lenses is shown in Fig. 2 on the left side.

V0: [0, 0°, 0, 90°, 0, 0, 0, 0] for objects at infinity
V0′: Add_Surface (V0, VTR, VR)
V1: Transform_Vergence (V0′,VD)
V1′ : Add_Surface (V1, VCA, VSIA, VCPA)
V2: Transform_Vergence (V1′, CCT/n_C_)
V2′ : Add_Surface (V2, VCP)
V3 : Transform_Vergence (V2′, (ELP-CCT)/n_A_)
V3′ : [n_V_/(AL_cor_-ELP), 0°, n_V_/(AL_cor_-ELP), 90°, 0, n_V_/(AL_cor_-ELP), 0, 0]

The required IOL power (IOLP) is extracted from VIOL = V3′–V3:

$$ \mathrm{VIOL}=\mathrm{Add}\_\mathrm{Surface}\ \left(\mathrm{V}3^{\prime },-\mathrm{V}3\right),\mathrm{and}\ \mathrm{the}\ \mathrm{sphere}\ \left(\mathrm{element}\ 1\right)\ \mathrm{or}\ \mathrm{equivalent}\ \mathrm{power}\ \left(\mathrm{element}\ 6\right)\ \mathrm{and}\ \mathrm{the}\ \mathrm{cylinder}\ \left(\mathrm{element}\ 5\right)\ \mathrm{in}\ \mathrm{an}\ \mathrm{axis}\ \left(\mathrm{element}\ 2\right)\ \mathrm{are}\ \mathrm{derived}\ \mathrm{from}\ \mathrm{vergence}\ \mathrm{VIOL}. $$

### Prediction of spectacle refraction after implantation of a tIOL

The calculation concept for the prediction of spherocylindrical spectacle refraction after implantation of a toric lens is displayed in Fig. 2 on the right side.

V3′ : [n_V_/(AL_cor_-ELP), 0°, n_V_/(AL_cor_-ELP), 90°, 0, n_V_/(AL_cor_-ELP), 0, 0]
V3 : AddSurface (V3′, -ViIOL)
V2′ : Transform_Vergence (V3, -(ELP-CCT)/n_A_)
V2 : AddSurface (V2′, -VCP)
V1′ : Transform_Vergence (V2, -CCT/n_C_)
V1: Add_Surface (V1′, -VCA, -VSIA, -VCPA)
V0′ : Transform_Vergence (V1, -VD)
V0 : [0, 0°, 0, 90°, 0, 0, 0, 0] for objects at infinity

The predicted refraction at the spectacle plane (PREF) is extracted from V0, VR, and V0′:

$$ \mathrm{VPREF}=\mathrm{Add}\_\mathrm{Surface}\ \left(\mathrm{V}0^{\prime },-\mathrm{V}0,-\mathrm{V}\mathrm{R}\right),\mathrm{and}\ \mathrm{the}\ \mathrm{sphere}\ \left(\mathrm{element}\ 1\right)\ \mathrm{or}\ \mathrm{equivalent}\ \mathrm{power}\ \left(\mathrm{element}\ 6\right)\ \mathrm{and}\ \mathrm{the}\ \mathrm{cylinder}\ \left(\mathrm{element}\ 5\right)\ \mathrm{with}\ \mathrm{the}\ \mathrm{axis}\ \left(\mathrm{element}\ 2\right)\ \mathrm{are}\ \mathrm{derived}\ \mathrm{from}\ \mathrm{the}\ \mathrm{vergence}\ \mathrm{VPREF}. $$

The applicability of this step-by-step approach is shown with 3 calculation examples: the first to calculate the power of a toric lens with curvature data from the corneal front and back surface, the second with curvature data from the corneal front surface and consideration of SIA and CPA, and the third to predict the refractive outcome at the spectacle plane in the situation of example 2 with a preset toric lens.

### Ethical approval

This article does not contain any studies with human participants or animals performed by any of the authors.

## Results

### Calculation example 1

For this 1st example, we have assumed that the corneal front (RCA_1_ = 7.9 mm/ACA_1_ = 10° and RCA_2_ = 7.6 mm) and back surface curvature (RCP_1_ = 6.8 mm/ACP_1_ = 20° and RCP_2_ = 6.6 mm) and the corneal thickness (CCT = 550 μm) are available from tomographic measurement. Axial length, anterior chamber depth, and lens thickness are measured as AL = 23.7 mm, ACD = 3.5 mm, and LT = 4.1 mm, respectively. With a toric lens implant, we wish to end up with a refraction at the spectacle plane (VD = 12 mm) with TRS = −0.1 dpt, TRC = −0.1 dpt at TRA = 90°. As formula constants, we used C = 0.424, H = −0.312 mm, and R = 0.077 dpt. Surgical-induced astigmatism was disregarded (SIAC = 0) in this example, and as we measured both corneal surfaces no correction for the corneal back surface astigmatism was necessary (CPAC = 0).

For the target refraction and corneal front and back surface, the respective vergences (without units) according to Eq. (1) read

VTR : [−0.10, 90, −0.20, 0, −0.10, −0.15, 0.10, 0.00]
VCA : [47.59, 10, 49.47, 100, 1.88, 48.53, 1.77, 0.64]
VCP : [−5.88, 20, −6.06, 110, 0.62, −5.97, −0.14, −0.11]

Table 2 summarizes the vergence in front of and behind all refractive surfaces as they are described in Fig. 1. The respective power of the IOL is derived from the difference of V3′ and V3 and yields IOLS = 19.32 and IOLC = 2.56 at IOLA = 99, and IOLEQ = 20.60, which refers to a spherical equivalent power of 20.60 dpt and an absolute cylinder power of 2.56 dpt with an implantation axis of 99°.

**Table 2:** Listing of vergences for example 1. Elements 1 to 8 refer to the elements of the vergence vector as described in Eq. (1), and the vergences V0 to V3′ are illustrated in Fig. 1. R/SIA/CPA correspond to the corrections for formula constant R, surgical-induced astigmatism, and corneal back surface astigmatism. As SIA and CPA were set to 0 in this example, V1 and V1 with SIA and CPA are identical. The power of the tIOL is calculated from the difference of vergences V3′ and V3 as marked in the table

Element ➔	1 in dpt	2 in °	3 in dpt	4 in °	5 in dpt	6 in dpt	7 in dpt	8 in dpt
Vergence↓								
V0 with R	−0.08	0	−0.08	90	0.00	−0.08	0.00	000
V0′	−0.28	0	−0.18	90	0.10	−0.23	0.10	0.00
V1	−0.28	0	−0.18	90	0.10	−0.23	0.10	0.00
V1 with SIA and CPA	−0.28	0	−0.18	90	0.10	−0.23	0.10	0.00
V1′	47.32	10	49.29	100	1.97	48.31	1.86	0.64
V2	48.23	10	50.29	100	2.05	49.26	1.94	0.67
V2′	42.34	9	44.23	99	1.89	43.29	1.80	0.55
V3	49.17	9	51.73	99	2.56	50.45	2.45	0.75
V3′	71.04	0	71.04	90	0.00	71.04	0.00	0.00

### Calculation example 2

For this 2nd example, we have assumed that the corneal front surface is measured and that the corneal back surface and central corneal thickness data are not available. Again, RCA_1_ = 7.9 mm/ACA_1_ = 10° and RCA_2_ = 7.6 mm, and from the fixed ratio of corneal front to back surface curvature of 7.77/6.4, we obtain RCP_1_ = 6.51 mm/ACP_1_ = 10° and RCP_2_ = 6.26 mm and CCT = 500 μm. For axial length, anterior chamber depth, lens thickness, and target refraction at spectacle plane, we have used the same values as in example 1. Again, the following values for formula constants were used: C = 0.424, H = −0.312 mm, and R = 0.077 dpt. In this example, the surgically induced astigmatism was set to SIAC = 0.20 dpt at incision axis 95°, and in the absence of tomographic measurements for the corneal back surface, a correction for corneal back surface astigmatism with CPAC = 0.27 at 90° has been used.

For the corneal back surface, the SIA, and the CPA correction, the respective vergences (without units) according to Eq. (1) read

VCP : [−5.88, 20, −6.06, 110, 0.62, −5.97, −0.14, −0.11]
VSIA : [−0.10, 95, 0.10, 5, 0.2, 0.00, −0.20, −0.03]
VCPA : [−0.14, 90, 0.14, 10, 0.27, 0.00, −0.27, 0.00]

Table 3 summarizes the vergences in front of and behind all refractive surfaces as described in Fig. 1. The respective power of the IOL is derived from the difference of V3′ and V3 and yields IOLS = 20.11 and IOLC = 1.83 at IOLA = 100, and IOLEQ = 21.03, which refers to a spherical equivalent power of 21.03 dpt and an absolute cylinder power of 1.83 dpt with an implantation axis of 100°.

**Table 3:** Listing of vergences for example 2. Elements 1 to 8 refer to the elements of the vergence vector as described in Eq. (1), and the vergences V0 to V3′ are illustrated in Fig. 1. R/SIA/CPA correspond to the corrections for formula constant R, surgical-induced astigmatism, and corneal back surface astigmatism. The power of the tIOL is calculated from the difference of vergences V3′ and V3 as marked in the table

Element ➔	1 in dpt	2 in °	3 in dpt	4 in °	5 in dpt	6 in dpt	7 in dpt	8 in dpt
Vergence↓								
V0 with R	−0.08	0	−0.08	90	0.00	−0.08	0.00	000
V0′	−0.28	0	−0.18	90	0.10	−0.23	0.10	0.00
V1	−0.28	0	−0.18	90	0.10	−0.23	0.10	0.00
V1 with SIA and CPA	−0.41	93	0.04	3	0.37	−0.23	−0.37	−0.03
V1′	47.55	12	49.07	102	1.52	48.31	1.40	0.61
V2	48.38	12	49.96	102	1.58	49.17	1.45	0.63
V2′	42.23	10	43.58	100	1.35	42.90	1.26	0.47
V3	49.10	10	50.93	100	1.83	50.01	1.72	0.64
V3′	71.04	0	71.04	90	0.00	71.04	0.00	0.00

### Calculation example 3

For this 3rd example, we predict the spectacle refraction after implantation of a tIOL. The data for corneal front surface curvature, SIA, CPA, AL, ACD, and LT from example 2 are used. Again, for corneal back surface, we have assumed a fixed front to back surface curvature ratio of 7.77/6.4, and for corneal thickness we use CCT = 500 μm. For the toric lens, we have assumed a spherical equivalent power of the tIOL with iIOLEQ = 21 dpt and an absolute cylinder power of iIOLC = 1.5 dpt implanted in an axis iIOLA = 105° (iIOLS = 20.25 dpt).

For the implanted lens, the respective vergence (without units) according to Eq. (1) reads

ViIOL = [20.25, 105, 21.75, 15, 1.50, 21.00, −1.30, −0.75]

Table 4 summarizes the vergence in front of and behind all refractive surfaces as they are described in Fig. 1. The respective power of the spectacle correction (residual refraction) is derived from the difference of V0″ and V0 with R and yields PREFS = −0.03, PREFC = 0.21 at PREFA = 97.5, and PREFEQ = 0.07, which refers to a refraction of −0.03 − 0.21 dpt/A 170° (minus cylinder) or −0.23 + 0.21 dpt/A 80° (plus cylinder).

**Table 4:** Listing of vergences for example 3. Elements 1 to 8 refer to the elements of the vergence vector as described in Eq. (1), and the vergences V0 to V3′ are illustrated in Fig. 1. R/SIA/CPA correspond to the corrections for formula constant R, surgical-induced astigmatism, and corneal back surface astigmatism. The refraction is predicted from the difference of vergences V0′ and V0 with R as marked in the table

Element ➔	1 in dpt	2 in °	3 in dpt	4 in °	5 in dpt	6 in dpt	7 in dpt	8 in dpt
Vergence↓								
V0 with R	−0.08	0	−0.08	90	0.00	−0.08	0.00	0.00
V0′	−0.31	80	−0.10	170	0.21	−0.21	−0.20	−0.08
V1	−0.31	80	−0.10	170	0.21	−0.21	−0.20	−0.08
V1 with SIA and CPA	−0.54	88	0.13	178	0.66	−0.21	−0.66	0.04
V1′	47.68	18	50.01	108	2.33	48.85	1.89	1.35
V2	48.52	18	50.93	108	2.41	49.73	1.96	1.40
V2′	42.37	15	43.48	105	1.10	42.92	0.96	0.55
V3	49.29	15	50.79	105	1.50	50.04	1.30	0.75
V3′	71.04	0	71.04	90	0.00	71.04	0.00	0.00

## Discussion

Today, there are several options available for correcting corneal astigmatism. In addition to classical spectacle correction and correction with soft or rigid contact lenses, there are surgical options such as corneo-refractive surgery in terms of PRK or LASIK, transverse or arcuate shaped corneal incisions using either a guided diamond knife or a femtosecond laser, toric phakic or add-on lenses for the phakic or pseudophakic eye, or toric capsular bag lenses implanted during normal cataract surgery. Spectacle correction with cylindrical lenses has the disadvantage of image distortion in terms of different magnifications in the principal meridians. Furthermore, if the spectacle correction is not perfectly aligned (decentration or tilt) or for peripheral vision, there are additional distortion effects such as coma, which may deteriorate the retinal image performance significantly [36]. In general, contact lenses induce fewer optical distortions compared to spectacles, but the proper alignment is not guaranteed.

Therefore, surgical procedures—if properly aligned—are beneficial for a stable correction of corneal astigmatism. Refractive procedures at the cornea are typically restricted to low or moderate cylinder values; e.g., corneal incisions allow correction of corneal astigmatism of up to 2.5 or 3 dpt. Excimer laser correction (PRK or LASIK) has good predictability and long-term stability of the refractive outcome for low and moderate cylinder values only. For larger corneal astigmatism values of 4 dpt up to 12 dpt and more, toric capsular bag or additional lenses yield a predictable option for refractive correction with a high patient satisfaction.

There are several options for calculation of toric lenses in clinical routine: more or less all manufacturers of toric lenses have developed online calculation features. Some of the online calculators require the biometric data for the eye and provide the spherical power (rarely) or spherical equivalent power (according to EN ISO 11979:2008) of the toric lens together with the absolute cylinder power and the implantation axis. Others require the spherical equivalent power of the lens and the corneal astigmatism as input data and derive the torus of the lens implant. In addition, some manufacturers offer a calculation service where the biometric data are entered in a calculation form and professional optometrists or opticians calculate the toric lenses individually. Furthermore, there are some manufacturer-independent calculation platforms such as the Barrett toric calculator where the biometric data are entered by the surgeon and various options for toric lenses (different lens models, different spherical/equivalent and torus options) are derived.

Calculating toric lenses with classical concepts such as standard formulae has strict limitations. If a toric lens calculation is split into 2 separate calculations of “spherical” lenses, we must take care that the estimated lens position does not depend on the corneal curvature. Otherwise, we obtain different effective lens positions, one for the flat and one for the steep meridian of the cornea, which does not make sense! This is at least true for the SRKT, the Hoffer-Q, and the Holladay1 formula [3–7, 9, 10]. In others, for example, the Haigis formula [12], the Olsen formula [8], or the Castrop formula [13], the effective lens position is not affected by corneal curvature.

If a sufficient number of tomographical data points are available, including either height or curvature data from the corneal front and back surface, the design data of the toric lens implant (front and back surface geometry and central thickness for all spherical and torus power steps and the refractive index), the pupil outline, and the positions of the cornea and toric lens relative to the visual axis, then full aperture ray tracing strategies are a powerful tool for toric lens calculation [19–22]. Ray tracing strategies overcome the limitations of linear Gaussian optics (with restrictions to the paraxial space) and in addition the asphericity of all refractive surfaces is considered [19, 20]. However, in many clinical situations, the relevant clinical data, such as corneal back surface data or the design data of the toric lens implant for all power steps, might be unavailable [19, 21].

Another task in toric lens power calculation is the surgical-induced astigmatism. More or less all calculation concepts offer the option to deal with an equivalent-neutral consideration of an astigmatic vector at the corneal front surface plane, which should consider the systematic vector change of corneal power due to cataract incision [22, 27]. This SIA should be customized to a specific surgeon and adapted to the surgical environment; therefore, a systematic evaluation of the preoperative to postoperative corneal shape in a sufficient number of cataracts performed previously is mandatory. The vector changes are normally processed in a double angle chart, and the centroid and confidence ellipse is calculated to differentiate between the systematic effect (SIA) and the stochastic scatter. If reliable data on the vector change are available, this SIA could be considered.

Another issue is the corneal back surface astigmatism. In the last decade, several papers have been published on corneal back surface astigmatism [24–26, 28–32]. If corneal or anterior segment tomography is available, we can directly extract data on the curvature of both corneal surfaces and central corneal thickness. In contrast, if we calculate a toric lens based on keratometry or corneal topography, we have to make assumptions regarding the corneal back surface. It is well known that using keratometry or topography to convert the radii in both principal meridians to corneal power does not properly reflect the situation of the cornea as a thick lens [8, 9, 16]. Different correction models have been developed to describe the deviation of the power of the astigmatic cornea from the keratometric values [23, 24, 28, 29]. Such corrections are typically considered an equivalent-neutral vector at the corneal front apex plane. In the present paper, we have considered this vector correction as CPA in case we do not have data on corneal back surface curvature and corneal thickness.

The present paper is based on the Castrop calculation strategy for spherical lenses. The Castrop formula is based on 3 formula constants. According to the Olsen formula, the C constant [17, 18] reflects the position within the crystalline lens where the haptic plane of the IOL implant will be located. This prediction strategy for the axial IOL position has been described previously in a basic form by Norrby et al. [15, 16]. Due to the different lens designs (optic and haptic design, material properties, axial shift of the optical part with respect to the haptic plane, etc.), an offset H is considered a second formula constant. The third constant (R) refers to an adjustment in refraction at the spectacle plane; e.g., it addresses the lane distance for refractometry. The cornea is considered thick lens with a corneal front surface and back surface curvature and central thickness. If tomographic data are available from both corneal surfaces and corneal thickness, they can be used directly in the calculation concept without restrictions of axis alignment. A correction for corneal back surface astigmatism is not necessary in such cases. If no data are available from corneal back surface curvature or thickness, the Castrop formula uses a preset value for the corneal thickness (500 μm) and derives corneal back surface curvature in both meridians from the respective corneal front surface curvature and a fixed proportion of front to back surface radius of 7.77/6.4 as derived from the Liou-Brennan schematic model eye [35]. In this case a correction for the corneal back surface curvature CPA should be applied at the corneal front apex plane. From the experience of some hundreds of eyes with toric lens implantation (PH), this correction is around 0.27 dpt in an axis 90°. Data from the Liou-Brennan schematic model eye are used for the refractive indices of the cornea, aqueous humor, and vitreous humor. A standard value of 12 mm is used for the vertex distance for the spectacle refraction.

The calculation concept as shown here is not restricted to any particular number of spherocylindrical surfaces. All surfaces and vector corrections are considered with cylinder axes oriented at random. Therefore, for calculation of a toric lens, the target refraction, the corneal front surface, and CPA or cornea front and back surface as well as SIA could act as crossed cylinders. The resulting toric lens power compensates all these cylinder values to obtain a plano vergence for V0 (for objects at infinity) or a spherical vergence V0≠0 (for objects at finite distances). For reverse calculation with any toric lens implanted in any orientation, this concept yields the predicted spherocylindrical refraction at the spectacle plane. The basic principle of this calculation concept has already been shown in previous papers.

The Castrop toric lens power calculation strategy is restricted to linear Gaussian optics (paraxial space). This simplification allows a straightforward calculation of the power of a toric lens or a prediction of spectacle refraction after implantation of any toric lens. If—instead of data such as sphere or cylinder or cylinder axis—the surface data of ALL refractive surfaces are available, a full aperture ray tracing strategy could be set up, which may yield more precise results for larger pupil sizes or for peripheral vision. However, such a full aperture ray tracing scheme is much more complex than any vergence- or matrix-based calculation concept. Another limitation of this study is that we restricted the model to a centered optical system with all surfaces aligned to the optical axis. Effects of decentration or tilt of refractive surfaces cannot be addressed with this calculation concept.

In conclusion, the Castrop formula for calculation of toric intraocular lenses and prediction of spectacle refraction of any toric lens is based on the Castrop formula for spherical lenses with 4 refracting surfaces. It uses spherocylindrical vergences throughout and can consider an arbitrary number of spherocylindrical surfaces with random cylinder axes. The effective lens position is derived from the phakic anterior chamber depth and lens thickness and 2 formula constants C and H, and a 3rd formula constant R is used to adjust the refraction at the spectacle plane. If tomographic data of both corneal surfaces are available, the Castrop formula considers the curvatures of both surfaces and corneal thickness. If corneal back surface data are unavailable, corneal thickness is preset and the back surface curvature is derived from front surface curvature using a fixed front to back surface curvature ratio, and a vector correction for back surface astigmatism could be included. Surgically induced astigmatism is considered a vector at the corneal front apex plane.

## Data Availability

All data generated or analyzed during this study are included in this published article.

## Code availability

Matlab Code on request
